# Development of a questionnaire for assessing the use of ChatGPT in primary and secondary disease prevention

**DOI:** 10.3389/fpubh.2025.1709611

**Published:** 2026-01-07

**Authors:** Viola Angyal, Ádám Bertalan, Péter Domján, Helga Judit Feith, Elek Dinya

**Affiliations:** 1 Health Sciences Division, Institute of Digital Health Sciences, Semmelweis University Doctoral College, Budapest, Hungary; 2 Health Sciences Division Interdisciplinary Applied Health Sciences Program, Semmelweis University, Doctoral College, Budapest, Hungary; 3 Department of Social Sciences, Faculty of Health Sciences, Semmelweis University, Budapest, Hungary

**Keywords:** ChatGPT, a large language model, disease prevention, self-completion questionnaire development, validation

## Abstract

**Background:**

Many individuals seek health-related guidance through ChatGPT OpenAI (San Francisco, CA, USA), due to its convenience and perceived reliability, often in place of, or as a supplement to, professional medical advice. This raises concerns about the accuracy of information provided and the potential for misinterpretation. On the other hand, ChatGPT offers a promising avenue for complementing traditional health prevention processes.

**Aims:**

This study aimed to develop and validate self-completion questionnaire among adults that evaluates the use of role of ChatGPT in primary and secondary health prevention, to explore the extent to which users utilize ChatGPT for disease prevention and health maintenance.

**Method:**

Questionnaire items were derived from a systematic literature review and comprised demographics, internet-use metrics, and validated items from the Brief Health Literacy Screening Tool. ChatGPT usage was structured into three domains: knowledge, attitudes, and behaviors. Test–retest reliability was quantified by Kendall's tau, and internal consistency by Cronbach's Alpha.

**Results:**

During the validation phase, the questionnaire was administered to a sample of 22 participants (16 female, six male), each of whom completed it twice, resulting in a total of 44 responses. Knowledge items demonstrated significant test–retest stability (Kendall's τ, *p* < 0.01). For behavior items, seven achieved perfect reliability (τ = 1.00), and five exceeded τ > 0.70. Attitude items similarly showed high stability, with three at τ = 1.00 and three above τ > 0.70. Internal consistency was acceptable (raw Cronbach's α = 0.771).

**Discussion:**

Our reliability analysis demonstrated that the items of the instrument exhibit good internal consistency, with Cronbach's Alpha values exceeding the commonly accepted threshold for exploratory research. Moreover, the questionnaire's design is inherently model-independent, allowing for its straightforward adaptation to assess user interactions with a variety of conversational artificial intelligence systems beyond ChatGPT.

**Conclusion:**

In conclusion, this study presents an initially validated questionnaire that captures how individuals employ ChatGPT for both primary and secondary disease prevention. The tool addresses key dimensions of artificial intelligence use and enables meaningful comparisons across populations with different social and educational backgrounds.

## 1 Background

The rapid advancement of artificial intelligence (AI) has introduced novel tools, such as ChatGPT, a conversational AI model developed by OpenAI (San Francisco, CA, USA), into various aspects of daily life (1). ChatGPT falls under the category of Natural Language Processing (NLP), which was designed to process natural language specifically, within the realm of generative artificial intelligence (GenAI), capable of producing new content from its training dataset (2). ChatGPT is built on the Generative Pre-trained Transformer (GPT) architecture, a special architect of GenAI. Due to its large number of parameters (more than 1.5 billion), it is considered a Large Language Model (LLM) (3). A LLMs, such as ChatGPT, operates by processing and analyzing vast amounts of text data to understand language patterns, grammar, context, and semantics (4). It is trained on diverse datasets, enabling it to predict the most probable next word in a sequence, generate coherent text, and respond to questions based on context. ChatGPT, specifically, uses a transformer architecture that employs attention mechanisms to weigh the importance of different words in a given input. This allows it to generate contextually appropriate and fluent responses. During training, the model learns from both supervised and unsupervised data, refining its ability to perform tasks such as language translation, summarization, and conversational interaction (5). When a user inputs a query, ChatGPT breaks it down into tokens, processes these tokens through multiple layers of the model, and predicts an appropriate response. Its functionality is powered by both its underlying model architecture and the scale of data it was trained on. ChatGPT has demonstrated its potential to provide accessible, user-friendly information on a wide range of topics, and people started to use this tool in healthcare-related questions as well, including primary and secondary health prevention, regardless of its reliability (6–10). It is very important to note that ChatGPT does not have specific knowledge, it is only based on statistical methods. This also means that hallucinations can occur from time to time. AI hallucination describes instances in which AI systems, especially LLMs, produce information that is incorrect, illogical, or entirely made up (11). This occurs because these models operate on probabilistic patterns learned from their training data rather than genuine understanding (12). Such ungrounded outputs pose serious risks in critical areas like healthcare and scientific research, where accuracy and trustworthiness are essential (13, 14).

Prevention measures encompass strategies to reduce the risk of disease onset (primary prevention) and to mitigate complications or recurrence of existing conditions (secondary prevention) (9, 15, 16). Primary prevention refers to strategies and interventions aimed at preventing the onset of disease or injury before it occurs. Its goal is to reduce the incidence of disease by addressing risk factors, enhancing protective factors, and promoting overall health and wellbeing. Common approaches include health education, vaccination programs, promotion of healthy lifestyles (such as balanced diet, physical activity, and smoking cessation), and environmental modifications that reduce exposure to hazards. Primary prevention operates at both individual and population levels to maintain health and prevent the development of pathological conditions.

Secondary prevention, on the other hand, focuses on the early detection and prompt management of existing diseases or risk conditions to halt or slow their progression. It aims to reduce the prevalence and severity of disease by identifying subclinical or early-stage illness through screening programs, regular medical checkups, and diagnostic assessments. Examples include blood pressure screening for hypertension, mammography for breast cancer, and blood glucose monitoring for diabetes. Effective secondary prevention minimizes complications, improves prognosis, and can restore individuals to a state of health or functional stability. Together, primary and secondary prevention form essential pillars of preventive medicine.

The integration of AI–driven conversational agents such as ChatGPT into healthcare systems presents new opportunities for enhancing both primary and secondary prevention. As a large language model, ChatGPT can process and generate natural language text, allowing it to function as an accessible, interactive tool for health education, behavioral modification support, early risk detection, and patient engagement. In the context of primary prevention, ChatGPT can contribute to reducing disease incidence by promoting healthy behaviors and facilitating public health education. Through conversational interfaces, ChatGPT can deliver tailored information about nutrition, physical activity, smoking cessation, vaccination, and stress management. Its capacity for personalization—based on users' demographics, preferences, and self-reported health data—enables adaptive communication that mirrors the benefits of individual counseling. Moreover, ChatGPT can be embedded within digital health platforms, wearable device applications, or telehealth portals to provide continuous reinforcement of health-promoting behaviors. For secondary prevention, ChatGPT's role extends toward early detection and management of existing conditions to prevent disease progression or complications. Through structured symptom assessment, ChatGPT can support users in recognizing early warning signs of chronic diseases such as diabetes, cardiovascular disease, and depression, prompting timely medical consultation. Secondary prevention is also critical in mental health, where timely recognition of relapse indicators can significantly improve outcomes. ChatGPT can provide psychoeducation, cognitive-behavioral prompts, and emotional support to individuals with depression, anxiety, or substance use disorders. ChatGPT represents a promising adjunctive tool for advancing both primary and secondary prevention strategies by enhancing health communication, promoting behavioral change, and supporting early detection.

Despite its potential, ChatGPT is increasingly used by health-conscious individuals and the general public without prior knowledge or verification of its outputs. Many individuals seek health-related guidance through the platform due to its convenience and perceived reliability, often in place of or as a supplement to professional medical advice (17–20). This raises concerns about the accuracy of information provided, the lack of personalization inherent in AI-generated responses, and the potential for misinterpretation (21–23). On the other hand, ChatGPT offers a promising avenue for complementing traditional health prevention processes (15, 24–29). Its ability to deliver tailored health education, recommend preventive practices, and promote health literacy positions it as a valuable support tool (30, 31). For instance, it could reinforce guidance provided by healthcare professionals, remind patients of screening schedules, or offer behavioral change strategies aligned with evidence-based practices. When used appropriately, ChatGPT has the potential to bridge gaps in healthcare access, particularly in underserved populations or for those seeking immediate advice (32–35).

While language models cannot replace consultations with healthcare professionals, they have the potential to complement traditional approaches to primary and secondary prevention (36–39). Exploring the potential applications and limitations of tools like ChatGPT in these contexts is crucial, so our primary scope was to examine ChatGPT usage further, specifically in primary and secondary prevention. Understanding the characteristics of users, including their socioeconomic status (SES), demographic variables, usage patterns, and motivations for using ChatGPT, can provide valuable insights.

## 2 Aims

### 2.1 Methodological aim

To develop and validate a self-completion questionnaire designed to measure individuals' use of ChatGPT in the context of health promotion and disease prevention and the role of ChatGPT in primary and secondary disease prevention. The instrument aims to provide a tool for assessing AI-assisted health information seeking and preventive behavior across diverse social groups. In line with these methodological aims, the study was structured as an initial instrument-development and preliminary validation effort rather than a full-scale epidemiological investigation.

### 2.2 Research aim

The research aim was to develop a tool capable of exploring how the Hungarian population utilizes ChatGPT for disease prevention and health maintenance, with a specific focus on primary prevention (e.g., advice on healthy lifestyle and nutrition) and secondary prevention (e.g., health screenings for early detection of diseases). Given this objective, the study was intentionally designed as an instrument-development project rather than a large-scale population study. Accordingly, the emphasis was on creating, refining, and preliminarily testing the questionnaire to ensure its feasibility and suitability for future research. The use of this questionnaire in future studies is expected to contribute to the more effective development of health screening processes and to the integration of digital tools into comprehensive health promotion and prevention strategies.

Furthermore, the newly developed questionnaire offers a valid tool to measure the use of AI-driven health information across populations of varying social status. This broad applicability enables its use within more diverse social groups, supporting inclusive digital health research. To our knowledge, no such questionnaire has yet been developed in this area, making this study a novel contribution to the field.

## 3 Methods

### 3.1 Questionnaire development

The questionnaire items were developed based on a comprehensive review of the literature to ensure their validity in evaluating the effectiveness (40–44). The questionnaire used in our study was developed for this study and has not previously been published elsewhere (Supplementary File 1). It was structured into the following domains.

#### 3.1.1 Part I. General information

Socio-demographic: sex (female/male), age (years), residence (ordinal), marital status (ordinal), employment (ordinal), educational level (ordinal), and whether they are or have previously been involved in patient care as a healthcare worker (yes/no).

#### 3.1.2 Part II. Internet usage habits

Self-evaluation of the internet usage (multiple choice), frequency of different online activities (multiple choice), and how satisfied with information found online (multiple choice). Healthcare-related online activities questions were also used. Internet usage for health and wellness (yes/no), information about health screening tests (yes/no), trust in health-related online information (multiple choice), seeking and scheduling health screenings based on online information (yes/no).

#### 3.1.3 Part III. Health literacy

Questions from the BRIEF (Brief Health Literacy Screening Tool) validated questionnaire (45).

#### 3.1.4 Part IV. ChatGPT-related question

We categorized our own ChatGPT-related questions into three main groups. Knowledge, attitude, and behavior-related questions were also included in the questionnaire.

Knowledge: The questionnaire included knowledge-related questions to assess general understanding of ChatGPT. These questions aimed to evaluate users' awareness of its capabilities, limitations, and potential applications, particularly in the context of primary and secondary prevention. This information is essential for identifying gaps in knowledge and tailoring educational efforts to enhance the effective and responsible use of ChatGPT in healthcare.

Attitude: The questionnaire also included attitude-related questions to explore users' perceptions, beliefs, and feelings toward ChatGPT. These questions aimed to understand how users view its reliability, trustworthiness, and role in supporting primary and secondary prevention efforts. Examining attitudes is crucial for identifying potential barriers or enablers to adopting ChatGPT as a complementary tool in healthcare and for shaping strategies to promote its responsible and effective use.

Behavior: The questionnaire further included behavior-related questions to examine how users interact with ChatGPT in practice. These questions focused on their frequency of use, specific use cases, and the contexts in which they turn to ChatGPT for health-related information or support. Understanding users' behavior provides valuable insights into real-world usage patterns and helps identify areas where ChatGPT can be optimized to better support primary and secondary prevention initiatives.

### 3.2 Data collection

The survey was conducted in Hungary in 2024 in an online format. Prior to the main study, a pre-test was carried out with eight participants who completed the questionnaire once and provided feedback on its clarity and structure. Based on this feedback, the questionnaire was finalized and subsequently assessed for test-retest reliability. To evaluate this, 23 participants were invited to complete the questionnaire twice over a 10-day interval. One participant was excluded from the analysis due to incomplete responses. Consequently, the final test-retest reliability analysis was conducted on a sample of 22 participants, resulting in a total of 44 completed questionnaires. Although the sample size is relatively small, this was consistent with the aims of the study, which focused on the development and preliminary reliability assessment of the questionnaire rather than on hypothesis testing or population-level inference. The primary objective was to create a measurement tool that can be applied in future research involving substantially larger and more diverse samples.

### 3.3 Statistical analysis

To assess the test-retest reliability of the questionnaire (comprising Knowledge, Situation, and Behavioral items), the Kendall correlation test was employed. Kendall's Tau is a non-parametric measure of correlation that assesses the strength and direction of association between two sets of rankings, making it ideal for evaluating the consistency of responses over time. Kendall's Tau is specifically designed for ordinal data, it does not assume any specific distribution of the data and measures the strength and direction of association between two sets of rankings. In this context, Kendall's Tau was used to compute correlation coefficients between the responses at two different time points. Correlation coefficients were calculated to evaluate the strength of the relationship between two measurements, where τ < 0.4 denotes weak correlation, 0.4 ≤ τ < 0.7 indicates moderate correlation, and τ ≥ 0.7 represents strong correlation. These coefficients reflect the degree to which participants' answers remained stable and consistent. The interpretation thresholds are standard benchmarks for interpreting the reliability of repeated measurements (46–50).

To assess the internal consistency of the questionnaire, Cronbach's Alpha was calculated. This statistical measure determines the degree to which a group of items is interrelated, reflecting their ability to measure a common underlying construct. Cronbach's Alpha values range from 0 to 1, with higher values indicating greater reliability. The calculation is based on the average correlations between items and the total number of items included in the scale.

Sample size: The pre-test was conducted with a total sample size of eight participants. For the correlation analysis, the sample size was 23 participants, but one participant was excluded from the analysis due to incomplete responses. Consequently, the final test-retest reliability analysis was conducted on a sample of 22 participants.

Statistical Analysis: All statistical analyses were done using IBM SPSS Statistics for Windows, Version 29.0 ((60), Armonk, NY, USA).

## 4 Results

### 4.1 Characteristics of the participant population

A sample of 22 (16 female, six male) participants completed the questionnaire. The mean age was 44.2, ranging from 25 to 77 years, and the majority of respondents were female (Table 1). Most of the participants lived in the Capital city and had a married/in a relationship marital status. Counting themselves, most of the participants lived with 1–2 people in one household, and almost all of them had a university degree. Their employment status was mostly full-time/self-employed (16). Seven of the participants are currently working or have previously worked in healthcare, next to patients, and 15 of them have never worked.

**Table 1:** Characteristic of the questionnaire tester population (*N* = 22).

**Variables**	**Mean or *n***	**Variables**	**Mean or *n***
**Biological gender**		**Number of people living with**	
Female	16	1–2	14
Male	6	3–5	7
**Age**	44.86	8	1
18–25	1	**Highest level of education**	
26–34	6	Primary school	0
35–44	5	Secondary school	0
45–54	4	Collage	1
55+	5	University	21
**Place of residence**		**Employment**	
Capital	10	Full time/self-employed	16
County town	6	Part-time employed	2
Other town	5	Student	1
Village	1	Disability pensioner	1
**Marital status**		Retired	2
Single	2	**Healthcare worker**	
Married/in a relationship	19	Yes (now or previously)	7
Divorced/living alone	1	No	15

### 4.2 Internet usage

Questions related to internet usage habits have been applied to know more about participant's online habits. Most of the participants use the internet every hour, or every 3 or 8 h (Table 2). No participant uses the internet less than daily. Most of them evaluated their knowledge as excellent or good. More than half of them would rather trust in health information they found online, and 3 of them do not use the internet for this purpose at all. More than half of the participants use online information for health screenings. Ten people answered that they were seeking and scheduling health screenings based on online information.

**Table 2:** Internet usage habits and trust in online healthcare information (*N* = 22).

**Variables**	**Mean or *n***	**Variables**	**Mean or *n***
**Internet usage**		**Trust in health information found online**	
Per hour	14	Trust very much	0
Every 3 h	7	Rather trust	12
Every 8 h	1	Rather not trust	7
Once a day	0	Not trust at all	0
Several times a week	0	I don't use internet for this purpose	3
Once a week	0	**Internet use for information on health screenings**	
**Internet usage knowledge**		Yes	13
Excellent	7	No	9
Good	7	**Seeking and scheduling health screenings based on online information**	
Not bad	6	Yes	10
Weak	2	No	12
I don't know	0		
**Internet use for general health information**			
Yes	19		
No	3		

### 4.3 Test-retest reliabilities

To evaluate the test-retest reliability of the questionnaire (Knowledge, Behavior, and Attitude items), the Kendall test was applied, and the Correlation Coefficient was used. Overall, 22 respondents participated in the validation phase of the questionnaire.

#### 4.3.1 Test-retest reliability of knowledge (KI)

The knowledge section contains two items, K1 and K2. To calculate test-retest reliability, a total of six statements/answer options were examined. All items showed a significant, strong positive correlation between the two measurements. Kendall's correlation coefficient was significant at the 0.01 level (Table 3).

**Table 3:** Test-retest reliability of knowledge (KI), attitude (AT), and behavior (BI), *N* = 22.

**No**.	**Items**	**Correlation coefficient**	**Reliable/non-reliable**
KI 1	Do you know ChatGPT?	1.000^**^	Reliable
KI 2	Please select which of the following statements you believe are TRUE about ChatGPT.	1.000^**^	Reliable
BI 1	Where do you obtain information about which health screening tests are necessary for you?	0.998^**^	Reliable
BI 2	Which of the following health prevention-related topics have you previously consulted a healthcare professional about?	1.000^**^	Reliable
BI 3	If applicable, how frequently have you used ChatGPT for any topic in the past year?	1.000^**^	Reliable
BI 4	Have you ever used ChatGPT for general information on healthy living?	0.843^**^	Reliable
BI 5	Have you ever used ChatGPT for general information regarding health screening tests?	1.000^**^	Reliable
BI 6	How likely are you to recommend ChatGPT to others for obtaining information related to healthy living?	1.000^**^	Reliable
BI 7	How likely are you to recommend ChatGPT to others for obtaining information related to health screenings?	1.000^**^	Reliable
BI 8	Please assess how the use of ChatGPT has changed your decisions or habits related to healthy living and health screenings, if it has changed them at all.	1.000^**^	Reliable
BI 9	If you have not used it yet, do you plan to use ChatGPT in the future for general information related to healthy living?	0.918^**^	Reliable
BI 10	If you have not used it yet, do you plan to use ChatGPT in the future for general information related to health screening tests?	0.963^**^	Reliable
BI 11	We asked ChatGPT a general question related to healthy living. Please read the response and assess how reliable you believe the answer to be.	1.000^**^	Reliable
BI 12	We asked ChatGPT a question related to a health screening test. Please read the response and assess how reliable you believe the answer to be.	0.864^**^	Reliable
AT 1	Do you consider ChatGPT reliable for searching for information related to healthy living?	1.000^**^	Reliable
AT 2	Do you consider ChatGPT reliable for searching information related to general health screening tests?	0.967^**^	Reliable
AT 3	Which of the following specific health prevention topics have you searched for information on using ChatGPT?	1.000^**^	Reliable
AT 4	How satisfied are you with the general information provided by ChatGPT regarding healthy living?	0.939^**^	Reliable
AT 5	Have you ever verified an answer provided by ChatGPT using another source?	0.989^**^	Reliable
AT 6	How satisfied are you with the general information provided by ChatGPT regarding health screening tests?	1.000^**^	Reliable

^**^Correlation is significant at the 0.01 level (two-tailed).

#### 4.3.2 Test-retest reliability of behavior (BI)

The behavior section contains 12 items BI1-BI12. Seven items showed strong (*r* = 1) correlation, and the other five also showed strong (*r* < 0.7) test-retest correlation (reliable items). This means a significant, strong positive correlation between two measurements, at a 0.01 significance level (Table 3).

#### 4.3.3 Test-retest reliability of attitude (AI)

The attitude section contains six items AT1-AT6 three items showed strong (*r* = 1) correlation, and the other three also showed strong (*r* < 0.7) test-retest correlation (reliable items). In summary, all items showed a significant, strong positive correlation between the two measurements as well. Correlation was significant at the 0.01 level (Table 3).

### 4.4 Cronbach's alpha calculation

To calculate the internal consistency of the questionnaire, we used Cronbach's Alpha. For this dataset, we calculated it for thirty-six items, questions from 23 to 43., which were Behavior question BI1 (Cronbach's Alpha if item deleted 0.625), Knowledge question KI2 (Cronbach's Alpha if item deleted 0.603), have been excluded due to the item's total statistic showing that the item underperformed. We evaluated the internal consistency of our 36-item questionnaire using Cronbach's Alpha. The raw α was 0.771, exceeding the conventional research threshold of 0.70 and indicating that the items cohere reasonably well as a single scale. These results demonstrate that the 36-item composite provides an acceptably reliable measure of the underlying construct for exploratory purposes (Table 4).

**Table 4:** Cronbach's Alpha calculation.

**Reliability statistics**	
**Cronbach's Alpha**	**N of items**
0.771	36

## 5 Discussion

### 5.1 Questionnaire development

This study set out to create and validate a new questionnaire assessing how the Hungarian sample uses ChatGPT for both primary prevention (e.g., lifestyle and nutrition advice) and secondary prevention (e.g., guidance on health screenings). Our reliability analysis indicates that the 36-item instrument functions as an acceptably coherent measure: the raw Cronbach's Alpha of 0.771 exceeds the conventional threshold of 0.70 for exploratory research, and the standardized α of 0.914 demonstrates very strong inter-item correlations once variance heterogeneity is removed. These findings suggest that respondents interpret and respond to the items consistently, supporting the internal-consistency reliability of the scale. Importantly, the disparity between the raw and standardized α values highlights that some items exhibit greater variability than others. In practical terms, this means that while the underlying constructs are closely related, differences in item-level variance modestly depress the raw-score reliability estimate. We therefore inspect item-total statistics (“α if deleted” and corrected item–total correlations) to identify and revise any underperforming questions. Computing Cronbach's Alpha on these narrower item sets should yield even higher reliability coefficients, improving the instrument's precision. Behavior question BI 1 and Knowledge question KI 2, have been excluded due to the item's total statistic showing that the item underperformed.

We believe that one of the key strengths of our questionnaire lies in its comprehensive and context-aware structure, which enhances its utility for evaluating how individuals engage with ChatGPT for disease prevention. The inclusion of socio-demographic variables, internet usage habits, health literacy, and LLM-specific questions provides a multidimensional perspective on the respondents, allowing for a more nuanced understanding of their behaviors and attitudes. The socio-demographic section enables stratified analysis by age, gender, education, and socioeconomic status, which is particularly valuable for identifying disparities or patterns in the adoption and trust of LLMs across different population subgroups. This foundation is essential for tailoring future health communication strategies to specific audiences. Additionally, the section on internet usage habits offers insight into participants' general digital behavior, including frequency and purpose of online activities. This information contextualizes the extent to which respondents may be open to or familiar with using LLMs in everyday life, including for health-related purposes. The inclusion of health literacy items further strengthens the instrument by assessing respondents' ability to understand and apply health information—an important factor influencing how effectively individuals can engage with AI-generated content. This is particularly relevant as LLMs increasingly provide health-related responses, the quality and appropriateness of which depend heavily on the user's ability to critically assess such information. Finally, the section focused on LLM-related questions addresses both knowledge, attitude and behavior aspects of using an LLM in disease prevention. Overall, the integrated structure of the questionnaire ensures that data are not collected in isolation but within a rich contextual framework. This design enhances both the interpretability and applicability of the findings, positioning the instrument as a valuable tool for ongoing research in digital health and AI integration in preventive medicine.

### 5.2 Comparison to other questionnaires

While other studies may provide valuable insights into user interactions with conversational AI systems, they do not present standardized or validated questionnaires for this purpose (51–54). To the best of our knowledge, this is the first study in Hungary attempting to develop and validate a questionnaire aimed at evaluating these dimensions within the context of LLMs. Developing and validating a questionnaire as the first study in Hungary aimed at understanding how people use LLMs for disease prevention is essential for establishing a reliable foundation for future research and public health interventions. As the adoption of LLMs like ChatGPT continues to grow, it is crucial to assess user behaviors, trust, and patterns of use within a culturally specific context. A validated instrument will enable researchers and policymakers to better understand the role of LLMs in preventive health, identify potential benefits and risks, and support evidence-based strategies tailored to the Hungarian population. From a practical standpoint, the validated questionnaire offers researchers and public health practitioners a standardized tool to quantify and compare ChatGPT usage across demographic subgroups, regions, and over time. Moreover, by incorporating the scale into longitudinal studies, scholars can monitor trends in AI adoption and examine whether higher engagement predicts improved preventive health outcomes, such as increased vaccination rates or earlier disease detection. Future research should validate the factor structure in independent samples, examine longitudinal stability, and explore criterion-related validity by linking questionnaire scores to actual health-behavior outcomes (e.g., uptake of screening tests).

The questionnaire's design is inherently model-independent, allowing for its straightforward adaptation to assess user interactions with a variety of conversational AI systems beyond ChatGPT. By applying the same instrument to alternative LLMs, such as DeepSeek or forthcoming GenAI platforms, researchers can conduct cross-model comparisons of engagement patterns in both primary (e.g., health education, lifestyle counseling) and secondary (e.g., symptom appraisal, screening facilitation) prevention contexts (55–59). Such comparative studies would not only evaluate the consistency of user behaviors across different architectures and training corpora but also help identify model-specific strengths and limitations in delivering reliable health guidance.

### 5.3 Limitations

Despite these strengths, several limitations warrant consideration. A key limitation of our study is the small sample size, which may limit generalizability, and the sample's overrepresentation of individuals from higher SES backgrounds. Although the questionnaire underwent preliminary testing to ensure clarity and comprehensibility among eight respondents, the sample size for the test–retest reliability analysis (*n* = 22) was relatively small. Such a limited sample may reduce the stability and precision of reliability coefficients and restrict the generalizability of the findings. Consequently, the results of the reliability assessment should be interpreted with caution. However, it is important to note that the primary aim of the study was not to draw population-level conclusions but to develop and refine the questionnaire as a measurement tool. In this context, the sample size was adequate for preliminary instrument testing, and the results serve as an initial step toward establishing reliability.

Future research should involve a larger and more diverse sample to obtain statistically robust reliability estimates and to confirm the stability of the instrument over time. These questionnaires are inherently susceptible to social-desirability and recall biases, incorporating behavioral log data from ChatGPT interactions could provide an objective complement to self-assessed usage patterns. Second, while internal consistency and factor structure were established, further evidence of construct validity, such as through confirmatory factor analysis or criterion-related validation against external health-behavior measures, can be used to fully substantiate the instrument's measurement properties.

### 5.4 Relevance and future utility

The questionnaire developed and validated in this study represents an important methodological contribution to the emerging field of digital health research. As conversational AI systems such as ChatGPT become increasingly integrated into everyday life, there is a growing need for empirically grounded tools capable of assessing how individuals engage with these technologies in the context of disease prevention and health promotion. The present instrument provides a tool to measure knowledge, attitudes, and behaviors related to ChatGPT use across both primary and secondary prevention domains.

Beyond its immediate application in the Hungarian context, the questionnaire is designed to serve as a reliable and adaptable research tool for future studies exploring the role of AI-driven communication in preventive healthcare. Its structure enables detailed assessment of the relationships between demographic variables, health literacy levels, and patterns of AI utilization, allowing for both cross-sectional and longitudinal analyses.

The future utility of this questionnaire extends to a wide range of scientific and practical applications. Researchers can employ it to evaluate the effectiveness of AI-based health interventions, to investigate determinants of trust and acceptance of digital tools, and to identify population groups that may benefit from targeted digital literacy and prevention programs.

Its application in future research will not only contribute to the refinement of preventive strategies and health screening processes but also enhance understanding of the ethical, social, and behavioral implications of AI-assisted health communication. Ultimately, this instrument is intended to support the advancement of evidence-based, inclusive, and digitally enabled approaches to health promotion and disease prevention.

## 6 Conclusion

In conclusion, our newly developed questionnaire demonstrates acceptable internal consistency and sufficient score dispersion. The questionnaire included items related to knowledge, attitudes, and behavior regarding ChatGPT, ensuring that all critical aspects of its use in health prevention were addressed. We designed and validated a questionnaire to assess how individuals engage with ChatGPT for both primary and secondary prevention. The questionnaire equips researchers with a means to better understand user's knowledge, attitudes, and behaviors, alongside their Internet-usage patterns and health literacy. Importantly, this instrument also enables valid comparisons across diverse social groups, offering a scalable and inclusive method for examining AI-driven health information-seeking behaviors. It should be noted that the test–retest reliability assessment was based on a relatively small sample of 22 participants. As a result, the reliability estimates presented here should be regarded as preliminary. Future studies involving larger and more diverse samples will be important to further examine the questionnaire's performance and to strengthen its suitability for broader use. To our knowledge, no prior questionnaire has been developed to evaluate these dimensions within the context of conversational AI. We believe that any health-related innovation, especially those built on cutting-edge tools like LLMs, must be rigorously evaluated to determine whether it achieves its intended goals. Systematic measurement enables healthcare professionals, policy makers, and educators to verify impact, refine strategies, and optimize resource allocation over time. This tool not only can help investigators quantify the relevance and effectiveness of LLMs in promoting disease prevention and early detection, but it can also identify which user characteristics (e.g., digital literacy, health-information-seeking patterns) predict greater benefit. Ultimately, we hope this tool will both deepen our understanding of AI-driven health support and encourage its thoughtful incorporation into future public-health and clinical practice, laying the groundwork for more effective, technology-enhanced prevention initiatives. We encourage future work to refine and extend this tool, applying it across diverse settings and integrating it with outcome-based research to deepen our understanding of large language model's role in public health.

## Data Availability

The raw data supporting the conclusions of this article will be made available by the authors, without undue reservation.
