# Supplementary material for: Development of a questionnaire for assessing the use of ChatGPT in primary and secondary disease prevention
Source: Front Public Health. 2026 Jan 7;13:1709611. doi: 10.3389/fpubh.2025.1709611 (PMC12819703; doi:10.3389/fpubh.2025.1709611)
Supplement: Supplementary file 1 [file Table_1.docx]

**Questionnaire**

**PART 1: Sociodemographic Questions**

1. Please indicate your age (in completed years).
2. Please indicate your gender.

☐ **Female**  ☐ **Male**  ☐ **Prefer not to answer**

1. **Where do you currently reside? (Please specify the location where you live on a regular basis.)**

☐ **Capital city**
☐ **County town**
☐ **Other town**
☐ **Village**

1. **Please indicate your current marital status.**

☐ **Married / In a relationship**
☐ **Widowed**
☐ **Single**
☐ **Divorced and living alone**
☐ **Other, please specify: __________**

1. Please indicate the number of individuals living in your household (including yourself).
2. Please indicate your highest level of education.

☐ **Primary school or less**
☐ **Vocational training school**
☐ **Technical school without a secondary school diploma**
☐ **Secondary school with diploma (e.g., high school, grammar school)**
☐ **College**
☐ **University**

1. Which of the following best describes your current employment status? (If multiple categories apply, please select the one that best represents your primary activity in terms of time commitment.)

☐ **Employed full-time**
☐ **Self-employed**
☐ **Employed part-time**
☐ **Unemployed (seeking work)**
☐ **Unemployed (not seeking work)**
☐ **Homemaker**
☐ **Full-time student/university student**
☐ **Retired**
☐ **Disability pensioner**
☐ **Other: __________________________**

1. Are you currently involved (or have you previously been involved) in patient care as a healthcare professional?

☐ **Yes**  ☐ **No**

**PART 2: Questions on Internet Usage**

1. How frequently do you use the internet?

☐ **Per hour**
☐ **Every 3 hours**
☐ **Every 8 hours**
☐ **Once a day**
☐ **Several times a week**
☐ **Once a week or less frequently**

1. How would you assess your level of internet proficiency?

☐ **Excellent**
☐ **Good**
☐ **Fair**
☐ **Weak**
☐ **I don't know**

3. **Do you use the internet to seek general information related to a healthy lifestyle (e.g., nutrition, physical activity, mental health)?**

☐ **Yes**  ☐ **No**

4. If you use the internet to seek general information related to a healthy lifestyle (e.g., nutrition, physical activity, mental health), how much do you trust the information you find online on these topics?

☐ **I trust it very much**
☐ **I rather trust it**
☐ **I rather do not trust it**
☐ **I do not trust it at all**

☐ **I do not use the internet to seek general information related to a healthy lifestyle.**

5. Do you use the internet to seek general information related to health screening examinations (e.g., chest X-rays, blood test results)?

☐ **Yes**  ☐ **No**

6. If you use the internet to seek general information related to health screening examinations (e.g., chest X-rays, blood test results), how much do you trust the information you find online regarding these screenings?

☐ **I trust it very much**
☐ **I rather trust it**
☐ **I rather do not trust it**
☐ **I do not trust it at all**

☐ **I do not use the internet to seek general information related to health screening examinations.**

7. Have you ever consulted a physician and scheduled a health screening examination (e.g., chest X-ray, blood tests) based on information you found on the internet?

☐ **Yes**  ☐ **No**

**PART 3: Brief Health Literacy Screening Tool (Mátyás et al., 2021)**

1. **How often do you need assistance in understanding written medical documents (e.g., outpatient records, discharge summaries, informed consent forms)?**

This question refers to documents written by a physician. If you do not usually visit a doctor or do not read the medical documents you receive, please select the 'I don't know' option.

☐ **Always**

☐ **Often**

☐ **Sometimes**

☐ **Very rarely**

☐ **Never**

☐ **I don't know**

2. **When seeking information about your health, how often do you find it difficult to understand patient information materials or brochures?**

This question refers to materials intended for laypersons, not professionals. If you do not usually read such materials, please select the 'I don't know' option.

☐ **Always**

☐ **Often**

☐ **Sometimes**

☐ **Very rarely**

☐ **Never**

☐ **I don't know**

**3. How often do you have difficulty understanding what is verbally communicated to you about your health?**

This question refers to information provided orally by a doctor, pharmacist, or other healthcare professional (e.g., nurse, public health nurse, physiotherapist). If you do not usually visit doctors or pharmacies, please select the 'I don't know' option.

☐ **Always**

☐ **Often**

☐ **Sometimes**

☐ **Very rarely**

☐ **Never**

☐ **I don't know**

4. **How confident do you feel when independently filling out forms related to your health condition?**

Please consider situations where you are required to provide written information about your own health (e.g., at a private medical practice, when purchasing insurance, or when accessing various healthcare services). If you have never filled out such forms, please select the 'I don't know' option.

☐ **Always**

☐ **Often**

☐ **Sometimes**

☐ **Very rarely**

☐ **Never**

☐ **I don't know**

**PART 4: ChatGPT-related questions**

**4.1. Knowledge (KI)**

KI 1.) Do you know ChatGPT?

☐ **Yes**  ☐ **No**

KI 2.) Please select which of the following statements you believe are TRUE about ChatGPT.

☐ **ChatGPT is a model based on artificial intelligence**
☐ **ChatGPT cannot replace a medical consultation**
☐ **ChatGPT possesses independent thinking abilities**
☐ **ChatGPT is capable of generating answers to user questions, engaging in dialogue, and conveying information**
☐ **ChatGPT is capable of always providing accurate medical diagnoses based on entered data, replacing medical consultation**
☐ **ChatGPT can assist in providing general health-related information**

**4.2. Behaviour (BI)**

BI 1.) Where do you obtain information about which health screening examinations (e.g., chest X-ray, blood tests) are necessary for you? (Multiple answers are possible)

☐ **General practitioner**
☐ **Invitation letter for population-based screening**
☐ **Social media (e.g., Facebook, Instagram, TikTok, etc.)**
☐ **Other online sources**
☐ **Friends/Family**
☐ **Other**
  **If you selected 'Other', please specify: __________**

BI 2.) Which of the following health prevention-related topics have you previously consulted a healthcare professional about? (Multiple answers are possible)

☐ **Questions related to healthy nutrition**
☐ **Questions related to proper physical activity**
☐ **Cervical cancer screening**
☐ **Breast cancer screening**
☐ **Colorectal cancer screening**
☐ **Chest X-ray screening**
☐ **Cardiovascular examinations**
☐ **Blood glucose testing**
☐ **Other blood tests**
☐ **Eye examination**
☐ **Other: __________**
☐ **I have not engaged in any health prevention activities at all**

BI 3.) If applicable, how frequently have you used ChatGPT for any topic in the past year?

☐ **Every hour**
☐ **Every 3 hours**
☐ **Every 8 hours**
☐ **Once a day**
☐ **Several times a week**
☐ **Once a week or less frequently**
☐ **I have not used ChatGPT**

BI 4.) Have you ever used ChatGPT for general information on healthy living **(e.g., nutrition, physical activity, mental health)?**?

☐ **Yes**  ☐ **No**

BI 5.) Have you ever used ChatGPT for general information regarding health screening tests (e.g., chest X-ray, blood tests)?

☐ **Yes**  ☐ **No**

BI 6.) How likely are you to recommend ChatGPT to others for obtaining information related to healthy living **(e.g., nutrition, physical activity, mental health)**?

☐ **Would highly recommend**
☐ **Would rather recommend**
☐ **Would rather not recommend**
☐ **Would not recommend at all**
☐ **I have not used ChatGPT**

BI 7.) How likely are you to recommend ChatGPT to others for obtaining information related to health screenings (e.g., chest X-ray, blood tests)??

☐ **Would highly recommend**
☐ **Would rather recommend**
☐ **Would rather not recommend**
☐ **Would not recommend at all**
☐ **I have not used ChatGPT**

BI 8.) Please assess how the use of ChatGPT has changed your decisions or habits related to healthy living and health screenings, if it has changed them at all.

☐ **Had a positive influence**
☐ **Neutral**
☐ **Had a negative influence**
☐ **Did not change it**
☐ **I have not used ChatGPT**

BI 9.) If you have not used it yet, do you plan to use ChatGPT in the future for general information related to health screening tests?

☐ **Definitely yes**
☐ **I’m rather planning to**
☐ **I’m rather not planning to**
☐ **Definitely not**
☐ **I am already using ChatGPT for this purpose**

BI 10.) If you have not used it yet, do you plan to use ChatGPT in the future for general information related to health screening tests?

☐ **Definitely yes**
☐ **I’m rather planning to**
☐ **I’m rather not planning to**
☐ **Definitely not**
☐ **I am already using ChatGPT for this purpose**

BI 11.) We asked ChatGPT a general question related to healthy living. Please read the response and assess how reliable you believe the answer to be.

**Question we posed to ChatGPT:**
"I would like to lose 4 kg over the next 3 months to improve my health. ChatGPT, please provide some dietary and exercise tips to help me lose weight, considering that I frequently experience back pain and have lactose intolerance."

**ChatGPT's response**

It's a great goal to strive for a healthier lifestyle, and losing 4 kg over the next 3 months is a reasonable and sustainable objective. Here are some dietary and exercise tips that can help you reach your goal, considering your back pain and lactose intolerance:

**Dietary Tips:**

1. **Balanced Diet**: Weight loss is based on a calorie deficit—consuming fewer calories than you burn. Focus on whole, nutrient-dense foods:
   - **Vegetables and fruits** at every meal—they’re low in calories but high in fiber and vitamins.
   - **Whole grains** such as brown rice, quinoa, and bulgur, which are rich in fiber and help you feel full longer.
   - **Proteins**: Increase your intake (e.g., chicken breast, fish, eggs, legumes), as protein helps preserve muscle mass, especially important during weight loss.
2. **Lactose-Free Options**: Since you are lactose intolerant, choose lactose-free dairy products (e.g., lactose-free yogurt, cheese, milk) or plant-based alternatives such as almond, coconut, or oat milk. These can also be good sources of protein and calcium.
3. **Healthy Fats**: Incorporate fats from sources like olive oil, avocados, nuts, and seeds. These help maintain satiety and reduce inflammation.
4. **Frequent Small Meals**: Eat 4–5 smaller meals a day to keep your blood sugar stable and avoid overeating. Be mindful of portion sizes.
5. **Avoid Processed Foods**: High-sugar and high-fat processed foods (e.g., fast food, pastries, soft drinks) tend to be calorie-dense and nutrient-poor.
6. **Stay Hydrated**: Aim to drink 2–2.5 liters of water daily. Hydration supports metabolism and weight loss.

**Exercise Tips for Back Pain:**

Since you have back pain, choose exercises that strengthen your back without overloading it. Always pay attention to pain signals and consult a doctor or physical therapist before starting any new routine. Here are some suitable options:

1. **Walking**: One of the best low-impact aerobic exercises that’s gentle on the back. Try to walk briskly for at least 30 minutes a day.
2. **Swimming or Water Aerobics**: These are full-body workouts that are gentle on the spine due to water buoyancy, which reduces joint and spinal load.
3. **Yoga and Pilates**: These help strengthen the back muscles and improve flexibility. They particularly enhance core strength, which supports your back.
4. **Strength Training**:
   - **Planks**: Great for strengthening the core, which can indirectly alleviate back pain.
   - **Upper Body Exercises**: Light dumbbell exercises (e.g., rows, bicep curls) help build back strength with minimal strain.
   - **Glute Bridges**: Strengthen the lower back and glute muscles.
5. **Stretching**: Always include thorough stretching before and after workouts, especially focusing on the back and leg muscles. This helps relieve tension and prevent pain.

**Other Important Tips:**

- **Sleep**: Sufficient and high-quality sleep (7–8 hours per night) is essential for both weight loss and managing back pain.
- **Stress Management**: Stress can contribute to back pain and weight gain. Include relaxation techniques in your routine, such as meditation or breathing exercises.

Make lifestyle changes gradually and be patient with yourself. Reaching your goal requires a long-term shift in habits, but it's a valuable path toward lasting health.

If you have further questions or need more specific advice, I'm happy to help!

How much do you trust the ChatGPT response provided above?

**☐ I trust it very much
☐ I rather trust it
☐ I rather do not trust it
☐ I do not trust it at all**

BI 12.) We asked ChatGPT a question related to a health screening test. Please read the response and assess how reliable you believe the answer to be.

**Question we posed to ChatGPT:**
"ChatGPT, please describe which health screening examinations are recommended for a healthy 28-year-old woman who performs sedentary computer-based work and has a family history of type 2 diabetes (mother and maternal grandmother)."

**ChatGPT's response**

For a 28-year-old woman performing sedentary computer-based work, it is important to participate regularly in certain screening examinations, especially considering a family history of type 2 diabetes. Regular screenings help detect diseases early, which is key to maintaining health. Here is a recommended list of screenings to consider:

### 1. ****Blood Glucose Testing****

Given that both the mother and maternal grandmother have type 2 diabetes, there is an increased hereditary risk. Regular blood glucose monitoring is recommended:

- **Fasting blood glucose**: Should be checked annually, especially if risk factors like family history and sedentary lifestyle are present.
- **HbA1c test**: Measures average blood glucose over the past 2–3 months. Annual testing is advisable if risk is elevated.

### 2. ****Blood Pressure Measurement****

High blood pressure often coexists with type 2 diabetes. Sedentary lifestyle contributes to hypertension risk:

- **Annual blood pressure check**. More frequent monitoring may be needed if family history includes hypertension.

### 3. ****Cholesterol Level Testing****

Monitoring cholesterol and triglycerides is important as elevated levels increase cardiovascular risk, especially with diabetes susceptibility:

- **Total cholesterol, LDL, HDL, and triglycerides**: Recommended every 2–3 years in young adults, or more frequently if abnormalities are detected.

### 4. ****Body Mass Index (BMI) and Waist Circumference****

Obesity is strongly linked to type 2 diabetes. Regular monitoring is advised:

- **BMI calculation**: Regularly assess body mass index.
- **Waist circumference**: Abdominal obesity poses a higher risk for cardiovascular diseases and diabetes.

### 5. ****Eye Examination****

Computer work strains the eyes and can lead to long-term visual problems:

- **Eye exam**: Recommended every two years to assess vision and eye health, especially due to screen exposure.

### 6. ****Musculoskeletal / Orthopedic Screening****

Sedentary work predisposes to musculoskeletal issues like back pain and neck strain:

- **Orthopedic assessment**: If symptoms are present, consult an orthopedist or physiotherapist. Regular physical activity and stretching can help prevent issues.

### 7. ****Gynecological Screenings****

- **Cervical cancer screening (Pap smear)**: Recommended every 3 years from age 21 if prior results were normal. With HPV testing, the interval may be extended to every 5 years.
- **Breast examination**: Monthly self-exams and clinical exams every two years are advised.

### 8. ****Vitamin D Level Check****

Lack of sun exposure from indoor work can lower vitamin D levels, affecting bone health, immunity, and overall well-being:

- **Vitamin D testing**: Annual check is advisable, especially if outdoor activity is limited.

### 9. ****Dermatological Screening****

Limited sun exposure and indoor work may affect skin health. Regular self-monitoring of moles is recommended:

- **Annual dermatological exam** for early detection of skin conditions or abnormalities.

### 10. ****Dental Check-up****

Oral health is essential for overall health:

- **Dental visit**: Recommended every 6–12 months for routine check-ups and cleaning.

### ****Summary:****

- Blood glucose and HbA1c: annually
- Blood pressure: annually
- Cholesterol: every 2–3 years
- Eye exam: every two years
- Regular gynecological screenings
- Monitoring of vitamin D, BMI, and waist circumference
- Orthopedic consultation if musculoskeletal symptoms occur

These screenings and self-assessments contribute to early disease detection and help maintain overall health, particularly in light of diabetes risk and the challenges associated with a sedentary lifestyle.

How much do you trust the ChatGPT response provided above?

**☐ I trust it very much
☐ I rather trust it
☐ I rather do not trust it
☐ I do not trust it at all**

**4.3. Attitude (AT)**

**AT 1.)** Do you consider ChatGPT reliable for searching for information related to healthy living?

☐ **Very reliable**
☐ **Rather reliable**
☐ **Rather unreliable**
☐ **Very unreliable**
☐ **I have not used ChatGPT**

**AT 2.)** Do you consider ChatGPT reliable for searching information related to general health screening tests?

☐ **Very reliable**
☐ **Rather reliable**
☐ **Rather unreliable**
☐ **Very unreliable**
☐ **I have not used ChatGPT**

**AT 3.)** Which of the following disease prevention-related topics have you previously consulted a healthcare professional about?

☐ **Questions related to healthy nutrition**
☐ **Questions related to proper physical activity**
☐ **Cervical cancer screening**
☐ **Breast cancer screening**
☐ **Colorectal cancer screening**
☐ **Chest X-ray screening**
☐ **Cardiovascular examinations**
☐ **Blood glucose testing**
☐ **Other blood tests**
☐ **Eye examination**
☐ **Other: __________**
☐ **I have used ChatGPT, but not in relation to health prevention**
☐ **I have not used ChatGPT**

**AT 4.)** How satisfied are you with the general information provided by ChatGPT regarding healthy living **(e.g., nutrition, physical activity, mental health)**?

☐ **Very satisfied**
☐ **Satisfied**
☐ **Not satisfied**
☐ **Very dissatisfied**
☐ **I have used ChatGPT, but not in relation to a healthy lifestyle**
☐ **I have not used ChatGPT**

**AT 5.)** Have you ever verified a response provided by ChatGPT using another source? If yes, please indicate from which of the following sources. (Multiple answers are possible)

☐ **Yes, on another website**
☐ **Yes, with a healthcare professional**
☐ **Yes, through another source. Please specify: __________**
☐ **I have not used ChatGPT**

AT 6.) How satisfied are you with the general information provided by ChatGPT regarding health screening tests (e.g., chest X-ray, blood tests)?

☐ **Very satisfied**
☐ **Satisfied**
☐ **Not satisfied**
☐ **Very dissatisfied**
☐ **I have used ChatGPT, but not in relation to a healthy lifestyle**
☐ **I have not used ChatGPT**

**References**

Mátyás, G., Vincze, F., & Bíró, É. (2021). Egészségműveltséget mérő kérdőívek validálása hazai felnőttmintán. *Orvosi Hetilap*, *162*(39), 1579-1588. <https://doi.org/10.1556/650.2021.32212>
